# Comparative safety and efficacy of 0.6 mg/kg versus 0.9 mg/kg alteplase in acute ischemic stroke: a systematic review and meta-analysis

**DOI:** 10.1186/s12883-025-04481-1

**Published:** 2025-11-18

**Authors:** Abdel Salam Ewais, Ahmad Alazzam, Mosab Said, Ahmad Alzyoud, Ahmad Dar Yassen, Saja Al-Shjrawi, Ayah Owaies

**Affiliations:** 1 Department of Family Medicine, Radiology and Emergency Medicine, Faculty of Medicine, the Hashemite University, Zarqa, Jordan; 2 Faculty of Medicine, The Hashemite University, Zarqa, Jordan; 3 Faculty of Medicine, Yarmouk University, Irbid, Jordan; 4 Faculty of Medicine, Jordan University of Science and Technology, Irbid, Jordan

**Keywords:** Stroke, Brain ischemia, Alteplase, Thrombolytic therapy, Intracranial hemorrhages

## Abstract

**Background:**

Alteplase is the standard thrombolytic therapy for acute ischemic stroke, with 0.9 mg/kg as the widely recommended dose. However, a lower dose of 0.6 mg/kg has been used, particularly in some populations, to reduce bleeding risk. We conducted a meta-analysis to compare the efficacy and safety of 0.6 mg/kg versus 0.9 mg/kg alteplase.

**Methods:**

A systematic search identified 11 observational studies and randomized controlled trials totaling 6,148 patients who received either low-dose or standard-dose alteplase within 4.5 h of symptom onset. Outcomes evaluated included symptomatic intracranial hemorrhage (sICH), any intracranial hemorrhage (ICH), 90-day mortality, in-hospital mortality, and functional outcomes measured by the modified Rankin Scale (mRS). Random-effects meta-analyses generated pooled odds ratios (OR) with 95% confidence intervals (CI).

**Results:**

Low-dose alteplase was associated with a significantly lower risk of sICH compared to standard-dose (OR 0.51, 95% CI 0.35–0.76, *p* = 0.0008). No significant differences were observed in any ICH (OR 1.00, 95% CI 0.84–1.19, *p* = 0.99), 90-day mortality (OR 0.86, 95% CI 0.71–1.04, *p* = 0.12), or functional independence defined by mRS 0–1 at 90 days (OR 0.90, 95% CI 0.80–1.01, *p* = 0.09). Heterogeneity was low to moderate across outcomes.

**Conclusion:**

Low-dose alteplase may reduce the risk of symptomatic hemorrhage without compromising mortality or functional recovery. These findings support consideration of dose individualization in clinical practice, warranting further prospective validation.

**Supplementary Information:**

The online version contains supplementary material available at 10.1186/s12883-025-04481-1.

## Introduction

Acute Ischemic strokes (AIS) are a leading cause of disability and death worldwide; timely treatment is essential to ensure optimal outcomes, in accordance with the principle that ‘time is brain [1]. Intravenous alteplase has been widely recognized as a method for the treatment of Acute Ischemic Strokes by thrombolysis at an effective dose of 0.9 mg per kilogram of body weight (10% as a bolus and the remaining 90% as infusion over 1 hour) [2]. However, the limited time window (4.5 hours from stroke onset), the high cost of the drug, and the risk of intracerebral hemorrhage are key factors limiting the widespread use of alteplase. Due to all the above reasons, the Japanese have introduced a new lower dose of alteplase [3]. The results of their trial led to the incorporation of the low dose into their clinical guidelines, which were the basis for widely adopting the low dose throughout Japan [4]. They also prompted further research into using the low dose.

In 2016, a large-scale trial (Enhanced Control of Hypertension and Thrombolysis Stroke Study) (ENCHANTED) was published. Although it did not show non-inferiority of the low dose, it did show that the low-dose patients experienced lower rates of intracranial hemorrhage [5]. These results, however, were inconsistent, as other observational studies showed non-inferiority [6, 7].

A definitive conclusion has not been reached, largely due to variability in study findings and contributing factors such as differences in population age, non-inferiority margins, and outcome measures [8]. As a result, the selection of the optimal dose remains unclear.

This meta-analysis aims to synthesize evidence on the efficacy and safety of 0.6 mg/kg versus 0.9 mg/kg alteplase in patients with acute ischemic stroke, focusing on outcomes including symptomatic intracranial hemorrhage (sICH), any intracranial hemorrhage (ICH), mortality (90-day and in-hospital), and functional outcomes measured by the modified Rankin Scale (mRS) at 90 days.

## Methods

Our study is a systematic review and meta-analysis methodology conducted according to the guidelines outlined in the Cochrane Handbook for Systematic Reviews of Interventions (PRISMA) [9]. The study protocol was registered in PROSPERO (CRD420251108674). The patient’s consent was not required due to the nature of the review.

### Search strategy

We conducted a comprehensive systematic search of PubMed, Scopus, and Web of Science databases from inception to October 2024, aiming to identify studies comparing low-dose (0.6 mg/kg) versus standard-dose (0.9 mg/kg) intravenous alteplase in acute ischemic stroke. The search combined controlled vocabulary terms and keywords related to “alteplase,” “thrombolysis,” “stroke,” “ischemic stroke,” and dosing regimens. No language restrictions were applied. Reference lists of relevant reviews and included studies were also screened manually to identify additional eligible articles. The detailed search strategies for each database are provided in the Supplementary file, Table 1.

**Table 1:** Summary of the included studies

Study ID	Year	Type of study	Country	Multi or single centric	Main inclusion criteria	Main exclusion criteria	Propensity Matched?
(Chen et al. 2022)	2022	Retrospective Cohort	Southeast China	Multi-center	Adults (≥ 18 years) with confirmed acute ischemic stroke; received IV alteplase (0.6 or 0.9 mg/kg) within 4.5 h of onset; complete baseline data available	(1) Patients with missing data regarding r-tPA dose or sICH information; (2) Those who received arterial thrombolytic treatment; (3) Those who underwent interrupted IVT.	Yes
(Yang et al. 2016)	2016	Retrospective Cohort	china	Single-center	Mild acute ischemic stroke; received rt-PA within 4.5 h of symptom onset; data prospectively recorded in a stroke registry (2013–2016)	Severe stroke, contraindications to thrombolytic therapy, pre-morbid mRS > 2, or loss to follow-up	No
(Hemasian et al. 2021)	2021	Retrospective Cohort	Iran	Single-center	patients older than 18 years old with ischemic stroke in brain computed tomography (CT), had rt-PA indications and received rt-PA within 270 min from starting their symptoms.	Typical alteplase contraindications (e.g., intracranial hemorrhage, recent surgery, bleeding risk), out of window (after 270 min)	No
(Chen* et al. 2022)	2022	Retrospective Cohort	Taiwan	Multi-center	Patients ≥ 18 years old with a clinical diagnosis of AIS, Patients in the treatment group received intravenous alteplase within a time window of 3–4.5 h	Typical rt-PA contraindications, Patients who received any other reperfusion therapy such as intra-arterial thrombolysis or endovascular thrombectomy	yes
(Škrbiü et al. 2019)	2019	Retrospective Cohort	Bosnia & Herzegovina	Single center	Acute ischemic stroke; age > 18 years; eligible for alteplase treatment per guidelines	Not available/not applicable	No
(Salem et al. 2021)	2021	Prospective Cohort	Egypt	Multi-center	age ≥ 18 years old, time window ≤ 4.5 h, patients presented with moderate to severe symptoms and demonstrate early improvement before starting alteplase, patients with seizures at time of onset if evidence suggests that residual impairment is secondary to stroke and not a postictal phenomenon, patients with blood pressure ≤ 185/110 or those who presented with high blood pressure and responded successfully to intravenous antihypertensive and patients with early ischemic changes)other than obvious hypodensity(as demonstrated on initial non contrast CT brain	Not available/not applicable	No
(Kim et al. 2018)	2018	Prospective cohort	Korea	Multi-center	Acute ischemic stroke; age ≥ 18 years; eligible for alteplase; treated within 4.5 h of onset	Not available/not applicable	No
(Sadeghi-Hokmabadi et al. 2021)	2021	Retrospective cohort	Iran	Multi-center	Patients registered at Iranian SITS centers; treated with IV thrombolysis	Missing data: alteplase dose, IVT status, or 3-month mRS not reported, centers with fewer than 20 patients	No
(Chao et al. 2019)	2019	Prospective Cohort	Taiwan	Multi-center	Age ≥ 80 years; NIHSS score > 4; door-to-needle time ≤ 3 h	Patients unlikely to benefit (e.g., severe disability or terminal illness), with interfering comorbidities, unable to adhere to follow-up, lacking consent, or previously enrolled in ENCHANTED	No
(Anderson et al. 2016)	2016	RCT	13 countries	Multi-centric	Men or women ≥ 18 years with acute ischemic stroke confirmed by imaging; treated within 4.5 h; prestroke mRS ≤ 1; systolic BP ≤ 185 mmHg (or lowered before treatment); no clear contraindication or preference for either alteplase dose	Patients who did not receive bridging therapy; typical contraindications to IV alteplase (e.g., hemorrhage, coagulopathy); absence of LVO or ineligibility for thrombectomy	No
(Mai et al. 2021)	2021	retrospective cohort	Vietnam	Single	Acute ischemic stroke due to large vessel occlusion; underwent mechanical thrombectomy after IV rt-PA	Retrospective exclusions: hemorrhagic stroke on baseline imaging; standard contraindications to thrombolysis	No

*AIS *acute ischemic stroke*, IVT* intravenous thrombolysis,* rt-PA *recombinant tissue plasminogen activator (alteplase),* sICH *symptomatic intracranial hemorrhage,* mRS *modified Rankin Scale*, IV *intravenous, *LVO *large vessel occlusion,* RCT *randomized controlled trial*. Studies varied in design*,* setting*,* inclusion/exclusion criteria*,* and whether propensity score matching was applied. “Not available/not applicable” indicates insufficient reporting in the source study. Chen2022 and Chen2022 refer to separate studies*

### Eligibility criteria

We included randomized controlled trials (RCTs) and cohort studies published in English that involved adult patients with acute ischemic stroke (AIS) treated with intravenous alteplase at either 0.6 mg/kg or 0.9 mg/kg within the standard 4.5-hour therapeutic window. Studies were required to report at least one of the following outcomes: sICH, any ICH, 90-day mortality, mRS scores of 0–1 or 0–2, in-hospital mortality, or full mRS distribution at 90 days. Studies were eligible for inclusion if they reported at least one primary outcome. We excluded non-English publications, case-control studies, case reports, case series, and cross-sectional designs. Studies were also excluded if they lacked a direct comparison between 0.6 mg/kg and 0.9 mg/kg alteplase, compared other doses or agents (e.g., tenecteplase), included patients treated outside the 4.5-hour window, or involved hemorrhagic stroke.

### Outcomes

The primary outcome were sICH, as defined within each included study. Definitions varied across trials and encompassed European Cooperative Acute Stroke Study II (ECASS II) [10], ECASS III [11], Safe Implementation of Thrombolysis in Stroke–Monitoring Study (SITS-MOST) [12], and National Institute of Neurological Disorders and Stroke rt-PA Stroke Trial (NINDS) [2] criteria, and any ICH, typically assessed by follow-up Computed tomography (CT) scan or magnetic resonance imaging (MRI) within 24–36 h of thrombolysis or at the time of clinical deterioration; Secondary outcomes included mortality, reported either as in-hospital or 90-day all-cause mortality; and functional outcomes, measured using the mRS. Functional outcomes were extracted both as dichotomized endpoints (mRS 0–1 and mRS 0–2 at 90 days) and as continuous data (mean mRS ± SD) when available. For each study, outcomes were collected separately for patients treated with low-dose alteplase and standard-dose alteplase. When multiple outcome definitions were reported, the most widely accepted or explicitly stated criterion was extracted. For all outcomes, when results from propensity-matched (PMS) analyses were available, these were prioritized over unadjusted outcomes to reduce confounding and provide a more accurate estimate of treatment effects.

## Data extraction

Data were extracted at three hierarchical levels: study-level, patient-level, and outcome-level. At the study, we collected the following information: study ID, publication year, study design (RCT or cohort), country of origin, whether the study was single-center or multi-center, primary inclusion and exclusion criteria, and whether propensity score matching was applied, as shown in Table 1. At the patient level, data were extracted separately for each treatment arm (0.6 mg/kg vs. 0.9 mg/kg alteplase), including sample size, proportion of female participants, mean or median age, and vascular risk factors (smoking, diabetes mellitus, hypertension, dyslipidemia, ischemic heart disease, atrial fibrillation, and prior stroke). We also extracted stroke onset-to-needle time (in minutes), baseline NIHSS score, and information on pre-treatment with antiplatelets or anticoagulants (type and dose). Stroke subtype based on TOAST classification (large artery atherosclerosis (LAA), cardioembolism (CE), small vessel occlusion (SVO), other determined etiology, or undetermined) as illustrated in Table 2. At the outcome level, we collected data on sICH, any ICH, 90-day mortality, mRS scores of 0–1 and 0–2 at 90 days, in-hospital mortality, and full mRS distribution at 90 days.

**Table 2:** Baseline characteristics of patients in the included studies

study id	Study arm	sample size	female	Age	smoking	DM	HTN	Dyslipidemia	IHD	Afib	Previous stroke	Onset-to-needle (min)	Baseline NIHSS score	Prior antiplatelet use	Prior anticoagulant use	LAA	CE	SVO	Undetermined	Other
(Chen et al. 2022)	Low-dose	88 (50%)	36 (40.9%)	68 (60–78)	NA	19 (21.6)	54 (61.4)	34 (38.6)	NA	29 (33.0)	14 (15.9%)	NA	7 (4–12)	NA	NA	NA	NA	NA	NA	NA
	Standard-dose	88 (50%)	26 (29.5%)	67 (61–72)	NA	23 (26.1)	67 (73.9)	42 (47.7)	NA	33 (37.5)	8 (9.1%)	NA	7 (4–12)	NA	NA	NA	NA	NA	NA	NA
(Yang et al. 2016)	Low-dose	46 (42.59%)	20, (43.48%)	65.5 (59–70)	NA	13 (28.3)	31 (67.4)	NA	4 (8.7)	5 (10.9)	NA	193.40 (56.09) Mean (SD)	4 (3–5)	NA	NA	12 (26.1%)	4 (8.7%)	16 (34.8%)	13 (28.8%)	1 (2.2%)
	Standard-dose	62 (57.41%)	28, (45.16%)	64.5 (59–72.25)	NA	21 (33.9)	46 (74.2)	NA	8 (12.9)	8 (12.9)	NA	190.21 (50.89) Mean (SD)	3 (2–5)	NA	NA	16 (25.8%)	5 (8.1%)	22 (35.5%)	17 (27.4%)	2 (3.2%)
(Hemasian et al. 2021)	Low-dose	187 (48.57%)	72, (38.51%)	68.38 ± 15.26	25 (13.36%)	59 (31.5)	105 (56.1)	33 (17.64)	22 (11.76)	12 (6.41)	18 (9.62%)	NA	10.89 ± 6.01	NA	NA	NA	NA	NA	NA	NA
	Standard-dose	198 (51.43%)	99 (50%)	69.20 ± 17.41	31 (15.65%)	47 (23.7)	101 (51.0)	35 (17.67)	29 (14.64)	23 (11.6)	15 (7.57%)	NA	10.45 ± 4.11	NA	NA	NA	NA	NA	NA	NA
(Chen* et al. 2022)	Standard-dose	65 (50%)	25 (38.5)	68 (59–74)	15 (23.1)	22 (33.9)	52 (80.0)	42 (64.6)	5 (7.7)	21 (32.3)	14 (21.5)	NA	10 (7–15)	16 (24.6)	1 (1.5)	15 (23.1)	20 (30.8)	12 (18.5)	NA	18 (27.7)
	Low-dose	65 (50%)	22 (33.8)	68 (59–74)	20 (30.8)	23 (35.4)	52 (80.0)	39 (60.0)	9 (13.9)	20 (30.8)	13 (20.0)	NA	10 (7–15)	20 (30.8)	2 (3.1)	19 (29.2)	19 (29.2)	10 (15.4)	NA	17 (26.2)
(Škrbiü et al. 2019)	Low-dose	45 (21.4%)	25 (55.6%)	67.47 ± 10.10	NA	28 (62)	NA	NA	NA	NA	NA	157 ± 55.17	10.48 ± 5.01	NA	NA	NA	NA	NA	NA	NA
	Standard-dose	165 (78.6%)	76, (46.1%)	61.88 ± 10.83	NA	14 (8.48)	NA	NA	NA	NA	NA	152.86 ± 53.50	11.93 ± 4.45	NA	NA	NA	NA	NA	NA	NA
(Salem et al. 2021)	Low-dose	40 (50%)	18 (45%)	60.58 ± 14.16	16 (40%)	19 (47.5)	26 (65)	14 (35)	12 (30)	12 (30)	5 (12.5%)	0–180; 4 (10%) 180–270; 36 (90%)	12.58 ± 4.37	12 (30%)	4 (10%)	14 (35%)	12 (30%)	18 (45%)	1 (2.5%)	0
	Standard-dose	40 (50%)	10 (25%)	61.03 ± 8.49	21 (52.5%)	12 (30)	26 (65)	15 (37.5)	13 (32.5)	6 (15)	4 (10%)	0–180; 39 (97.5%) 180–270; 1 (2.5%)	14.03 ± 4.17	3 (7.5%)	2 (5%)	9 (22.5%)	7 (17.5%)	14 (35%)	4 (10%)	1 (2.5)
(Kim et al. 2018)	Low-dose	178 (50.7%)	56, (31.5%)	65 (57–72)	61 (34%)	40 (23)	94 (53)	27 (15)	17 (10)	29 (16%)	18 (10%)	113 (88–171)	7 (4–13)	57 (32%)	9 (5%)	65 (39%)	50 (30%)	26 (16%)	24 (14%)	3 (2%)
	Standard-dose	173 (49.3%)	57, (32.95%)	66 (60–73)	55 (32%)	34 (20)	95 (55)	29 (17)	19 (11)	28 (16)	21 (12%)	115 (84–165)	8 (5–12)	37 (22%)	7 (4%)	56 (35%)	51 (32%)	26 (16%)	26 (15%)	2 (1%)
(Sadeghi-Hokmabadi et al. 2021)	Low-dose	149 (14.1%)	77, (51.6%)	77 (63.0–82.5.0.5)	8 (5.3%)	29 (19.4)	117 (78.5)	32 (21.4)	0	32 (21.4)	0	53 ± 6	11 (6.2–18)	50 (33.5)	7 (4%)	NA	NA	NA	NA	NA
	Standard-dose	906 (85.9%)	382, (42.2%)	68 (57.0–78.0)	103 (11.3%)	204 (22.5)	642 (70.8)	136 (15)	29 (3.2)	118 (13)	21 (2.3%)	56 ± 13	10 (6–16)	363 (40%)	43 (4.7%)	NA	NA	NA	NA	NA
(Chao et al. 2019)	Low-dose	108 (43.3%)	59 (54.6%)	83 (81–87)	19 (17.6%)	31 (28.7%)	89 (82.4%)	27 (25)	19 (17.6)	59 (54.6)	NA	134.4 ± 46.5	14.6 ± 6.3	25 (23.2%)	1 (0.93%)	NA	NA	NA	NA	NA
	Standard-dose	141 (52.7%)	66 (46.8%)	82 (80–85)	30 (21.3%)	43 (30.5)	113 (80.1)	61 (43.3)	21 (14.9)	69 (48.9)	NA	136.1 ± 47.3	14.0 ± 6.7	30 (21.3%)	4 (2.8%)	NA	NA	NA	NA	NA
(Anderson et al. 2016)	Low-dose	1654 (50.16%)	634 (38.33%)	68 (58–76)	377 (22.9%)	325 (19.7)	1031 (62.6)	297 (18)	256 (15.5)	330 (20.1)	287 (17.4%)	170 (125–218)	8 (5–14)	407 (24.7%)	48 (2.9%)	622 (38.3%)	324 (19.9%)	334 (20.6%)	NA	295 (18.2%)
	Standard-dose	1643 (49.94%)	614 (37.37%)	67 (58–76)	393 (24%)	321 (19.6)	1034 (63)	258 (15.7)	223 (13.6)	306 (18.7)	302 (18.4%)	170 (127–219)	8 (5–14)	345 (21.1%)	34 (2.1%)	648 (40.3%)	317 (19.7%)	339 (21.1%)	NA	258 (16.1%)
(Mai et al. 2021)	Low-dose	73 (68.22%)	27 (36.99%)	62.4 ± 11.4	18 (24.7%)	6 (8.2)	19 (26)	2 (2.7)	NA	9 (12.3)	NA	206.3 ± 47.9	16 (14–19)	0	0	42 (57.5%)	20 (27.4%)	NA	11 (15.1%)	NA
	Standard-dose	34 (31.78%)	13 (38.24%)	63.6 ± 11.8	11 (32.4%)	3 (8.8)	15 (44.1)	2 (5.9)	NA	2 (5.9)	NA	202.2 ± 53.8	14 (11–16)	0	1 (2.9%)	17 (50%)	10 (29.4%)	NA	7 (20.6%)	NA

*DM *diabetes mellitus,* HTN *hypertension,* IHD *ischemic heart disease,* Afib *atrial fibrillation,* NIHSS *National Institutes of Health Stroke Scale,* LAA *large artery atherosclerosis,* CE *cardioembolism,* SVO *small vessel occlusion*.* “Study arm” indicates the treatment group (e.g., 0.6 mg/kg vs. 0.9 mg/kg alteplase). Values are reported per arm when available. “Onset-to-needle” refers to time in minutes from stroke symptom onset to IV thrombolysis. Stroke subtypes (LAA, CE, SVO, Undetermined, Other) are classified according to TOAST criteria were reported. Missing or unreported variables are marked accordingly in the data tables.

### Statistical analysis

We used Mantel-Haenszel odds ratios (OR) for dichotomous outcomes (sICH, any ICH, 90-day mortality, In-hospital mortality, mRS 0–1, and mRS 0–2) with a Random-effects model. For overall mRS (continuous outcome), we used mean difference (MD) with a random-effects model to account for potential variability in mRS reporting. Heterogeneity was assessed using I² statistics. Analyses were performed in Review Manager (RevMan) 5.4.1. A p-value < 0.05 was considered significant. We also conduct.

### Subgroup analysis

To further explore sources of variation in sICH, we conducted a predefined subgroup analysis stratified by the diagnostic criteria used in the original studies (NINDS, ECASS II/III and SITS-MOST). Studies were grouped according to the specific definition of sICH applied, and pooled effect estimates were calculated separately for each subgroup using a random-effects model.

### Risk of bias

We assessed study quality using the Cochrane Risk of Bias tool for RCTs and the Newcastle-Ottawa Scale for observational studies. The Newcastle Ottawa scale (NOS) for quality assessment of non-randomized trials [13] was used for the quality assessment of the included studies. The NOS assigns a maximum of nine points for the three domains: [1] Selection of study groups (four points); [2] Comparability of groups (two points); and 3) Ascertainment of exposure and outcomes (three points). NOS’s total score of 0 to 3 indicates a high risk of bias, 4 to 6 indicates a moderate risk, and ≥ 7 indicates a low risk of bias. For randomized controlled trials, we used the Cochrane Risk of Bias 2 (RoB 2) tool [14], which evaluates five domains: randomization process, deviations from intended interventions, missing outcome data, measurement of the outcome, and selection of the reported result. Judgments were made as “low risk,” “some concerns,” or “high risk” according to standard RoB 2 criteria.

### Strength of evidence

We assessed the strength of evidence using the GRADE system. It looks at factors like study design, quality, consistency, directness, and precision. GRADE rates evidence as high, moderate, low, or very low. High means further research is unlikely to change the result, while very low means we are very uncertain about the effect.

## Results

### Search results

**Fig. 1:**
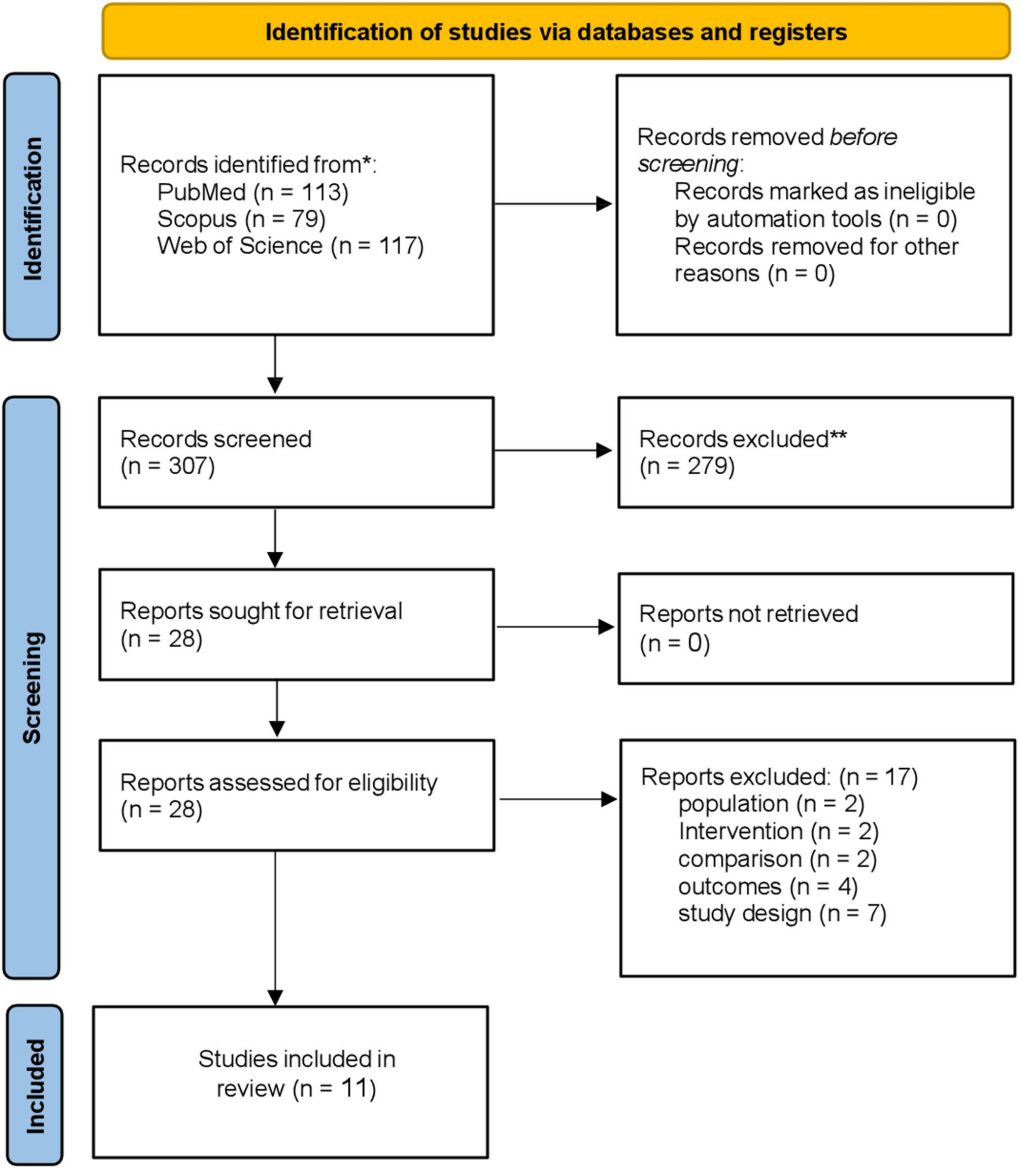
PRISMA flowchart

A total of 307 records were identified through electronic databases, including PubMed (*n* = 113), Scopus (*n* = 79), and Web of Science (*n* = 117). All 307 records underwent title and abstract screening, of which 279 were excluded. The remaining 28 full-text reports were retrieved and assessed for eligibility. Seventeen reports were excluded for the following reasons: population mismatch (*n* = 2), intervention or comparator issues (*n* = 4), outcome not reported (*n* = 4), and inappropriate study design (*n* = 7). Ultimately, 11 studies met the inclusion criteria and were included in the final systematic review and meta-analysis. The full selection process is detailed in Fig. 1 (PRISMA flow diagram).

### Study characteristics

We included eleven studies, comprising one randomized controlled trial [5] and ten observational cohorts divided into 7 retrospective [15–21] and 3 prospective [6, 7, 22] studies. These studies, published between 2016 and 2022, collectively evaluated 6,148 adult patients with AIS treated with intravenous alteplase. Of these, 2,633 (36.3%) received a low-dose (0.6 mg/kg) regimen and 3,515 (57.1%) received the standard-dose (0.9 mg/kg).

Studies were conducted across a diverse range of global healthcare settings, including six studies in Asia (China, Taiwan, Korea, Vietnam) [6, 7, 15, 17, 19, 20], three studies in the Middle East (Iran, Egypt) [18, 21, 22], one study in Europe (Bosnia and Herzegovina) [16], and a multinational RCT spanning 13 countries [5]. Seven studies were Multicentric [5–7, 17, 20–22], and two studies [17, 20] employed PMS. The primary Inclusion criteria typically required imaging-confirmed AIS and eligibility for intravenous thrombolysis within a time window of 3 to 4.5 h from symptom onset. Exclusion criteria varied but generally followed standard alteplase contraindications (e.g., intracranial hemorrhage, recent surgery, coagulopathy, or pre-stroke disability). Study characteristics and baseline patient demographics are provided in Tables 1 and 2.

The mean age of participants ranged from approximately 61 to 83 years, with the oldest cohorts featured in studies specifically enrolling octogenarians [6]. The proportion of female participants varied from 25% to 54.6%. Cardiovascular risk factors were highly prevalent; hypertension was reported in 44.1% to 82.4% of patients, diabetes mellitus in 8.2% to 47.5%, and atrial fibrillation in 5.9% to 54.6%. The median NIHSS score, a measure of stroke severity, ranged from 3 to 16, indicating that the studies included patients with a wide spectrum of stroke severity, from minor to severe. The mean onset-to-needle time was generally within the therapeutic window, ranging from approximately 113 to 206 min, as illustrated in Table 3.

**Table 3:** Overview of reported clinical outcomes across included studies

Study	Dose (mg/kg)	sICH (event/total)	Definition of sICH	Any ICH (event/total)	Definition of Any ICH	90-Day Mortality (event/total)	In-Hospital Mortality (event/total)	mRS 0–1 (event/total)	mRS 0–2 (event/total)	Mean mRS (mean ± SD)
Chen2022*	0.6	1/65	ECASS III	9/65	CT/MRI performed 24–36 h after thrombolysis.	NR	NR	24/65	56/65	2.65 ± 1.89
	0.9	2/65		10/65				22/65	57/65	2.52 ± 1.88
Chao2019	0.6	4/108	*SITS-MOST* and NINDS	NR	NR	10/108	NR	15/108	24/108	3.7 ± 1.6
	0.9	5/141		NR		19/141	NR	32/141	49/141	3.3 ± 2.0
Mai2021	0.6	4/73	ECASS II	19/73	Not reported	2/73	NR	NR	50/73	NR
	0.9	4/34		11/34		3/34	NR	NR	22/34	
Chen2022	0.6	2/88	ECASS II	14/88	CT-visible ICH, regardless of neurological decline.	NR	0/88	NR	NR	NR
	0.9	10/88		18/88			3/88			
Kim2018	0.6	6/178	NINDS	22/178	CT or MRI conducted at baseline, within 24 h post-procedure, and at the time of clinical deterioration.	4/178	4/178	82/178	110/178	2.14 ± 1.6
	0.9	14/173		27/173		5/173	5/173	82/173	106/173	2.18 ± 1.61
Salem2021	0.6	0/40	ECASS III	1/40	Non-contrast CT brain scans performed 24 h post-treatment.	2/40	1/40	NR	25/40	NR
	0.9	3/40		5/40		1/40	1/40	NR	25/40	
Sadeghi2021	0.6	3/149	SITS-MOST	NR	NR	29/149	NR	72/149	78/149	2.63 ± 2.55
	0.9	14/906				145/906		488/906	558/906	2.31 ± 2.44
Yang2021	0.6	2/46	ECASS III	NR	NR	NR	NR	34/46	12/46	1.0 ± 1.53
	0.9	3/62				NR		44/62	18/62	0.67 ± 1.52
Anderson2019	0.6	17/1654	*SITS-MOST*, NINDS, ECASS II and ECASS III	277/1654	CT or MRI at 24–36 h post-treatment, per usual clinical practice.	140/1654	752/1607	34/46	1002/1607	2.13 ± 1.91
	0.9	35/1643		294/1643		170/1643	782/1599	44/62	1007/1599	2.16 ± 1.81
Škrbiü2019	0.6	NR	NR	6/45	follow-up CT scans.	0/45	NR	NR	24/45	2.2 ± 1.91
	0.9			35/165		10/165			106/165	2.11 ± 2.11
Hemasian2021	0.6	NR	NR	18/187	NR	8/187	19/187	NR	NR	2.27 ± 1.94
	0.9			19/198		9/198	17/198			1.94 ± 1.83

*sICH* symptomatic intracranial hemorrhage, *ICH* intracranial hemorrhage, *mRS* modified Rankin Scale, *NR* not reported, *CT/MRI* computed tomography/magnetic resonance imaging, *ECASS II/ECASS III* European Cooperative Acute Stroke Study II/III criteria for sICH, *SITS-MOST* Safe Implementation of Thrombolysis in Stroke-Monitoring Study criteria for sICH, *NINDS* National Institute of Neurological Disorders and Stroke criteria for sICH, Any ICH: CT- or MRI-confirmed hemorrhage, may include only symptomatic events; 90-day mortality: all-cause mortality at 90 days post-thrombolysis; In-hospital mortality: death during hospitalization; Dose: alteplase administered in mg/kg (low 0.6, standard 0.9); mean mRS reported as mean ± SD.

Regarding stroke etiology, classified according to the Trial of Org 10,172 in Acute Stroke Treatment (TOAST) criteria [23], were available for six of the eleven included studies [5, 7, 15, 17, 19, 22]. The most prevalent etiology was large artery atherosclerosis (LAA), representing between 22.5% and 57.5% of patients in the reported arms. Cardioembolism (CE) was also a major contributor, accounting for

 8.1% to 30.8% of strokes. Small vessel occlusion (SVO) was reported in a significant proportion of patients (15.4% to 45.0%), particularly in the study by Salem et al. (2021) [22]. A substantial number of strokes were classified as “undetermined” etiology (up to 28.8%) or “other” determined etiology (up to 27.7%).

There was variability in the definitions used for sICH, with studies employing various criteria: ECASS II [5, 15, 20], ECASS III [5, 17, 19, 22], SITS-MOST [5, 6, 21], and NINDS [5–7]. The reported incidence of sICH varied across nine studies [5–7, 15, 17, 19–22] through 5,553 patients, while eight studies [5, 7, 15–18, 20, 22] assessed any ICH across 4,736 patients. The definitions and frequencies of all outcomes are detailed in Table 3.

### Primary outcomes

**Fig. 2:**
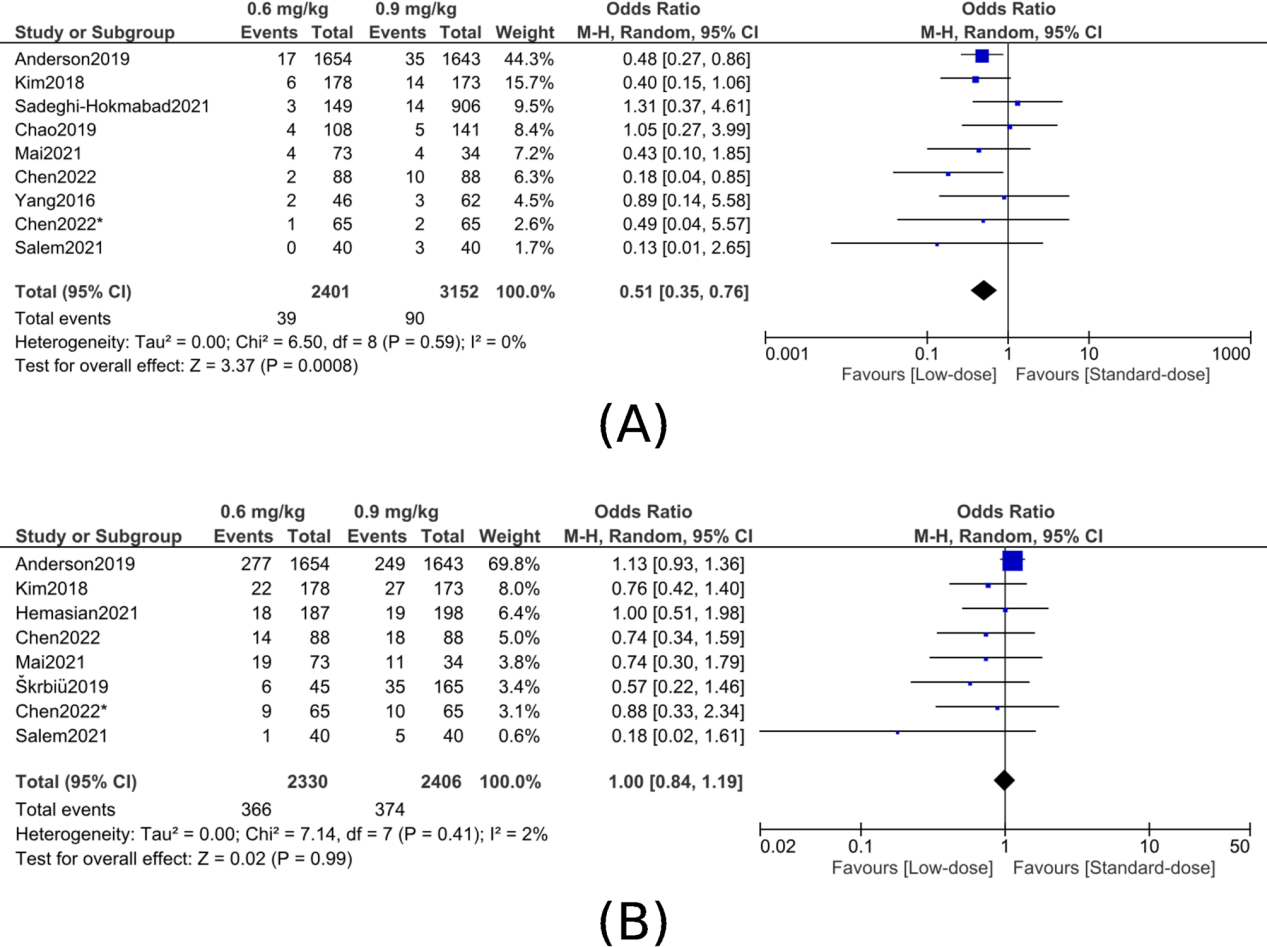
Comparison of low-dose (0.6 mg/kg) versus standard-dose (0.9 mg/kg) alteplase for intracranial hemorrhage outcomes. **A**, Symptomatic intracranial hemorrhage (sICH). **B**, Any intracranial hemorrhage (any ICH)

A. sICH:
  A. In this meta-analysis of 5,553 patients, treatment with low-dose was associated with approximately a 49% reduction in the odds of sICH compared with standard-dose (OR, 0.51; 95% CI, 0.35–0.76; P = 0.0008), with no heterogeneity observed across studies (I² = 0%) (figure 2A). Subgroup analyses according to sICH definitions revealed variable treatment effects when compared to standard-dose therapy. Low-dose alteplase was linked to a non-significant decrease in sICH risk according to SITS-MOST criteria (OR, 0.69; 95% CI, 0.36–1.33; P = 0.27; I2 = 26%). Low-dose therapy significantly decreased the risk of sICH for ECASS II criteria (OR, 0.52; 95% CI, 0.30–0.88; P = 0.02; I² = 18%). Similarly, low-dose alteplase significantly reduced the odds of sICH under ECASS III criteria (OR, 0.47; 95% CI, 0.29–0.78; P = 0.003; I² = 0%). On the other hand, there was no difference for NINDS criteria (OR, 0.73; 95% CI, 0.43–1.24; P = 0.24; I² = 39%). Statistical tests revealed no significant differences between definitions across subgroups (P for interaction = 0.61; I2 = 0%), indicating that low-dose alteplase reduces the risk of sICH in a generally consistent manner, though it varies depending on the definition used (Figure S1).

B. Any ICH:
  Across all studies, there was no significant diffence in the risk of any ICH between low- and standard-dose alteplase (OR, 1.00; 95% CI, 0.84–1.19; P = 0.99; I² = 2%) (Figure 2B).

### Secondary outcomes

1. Mortality
  A 90-day mortality: Eight studies (5,734 patients) showed no significant difference in 90-day mortality between low- and standard-dose alteplase (OR, 0.86; 95% CI, 0.71–1.04; P = 0.12), with no heterogeneity observed (I² = 0%) (figure 3A).

**Fig. 3:**
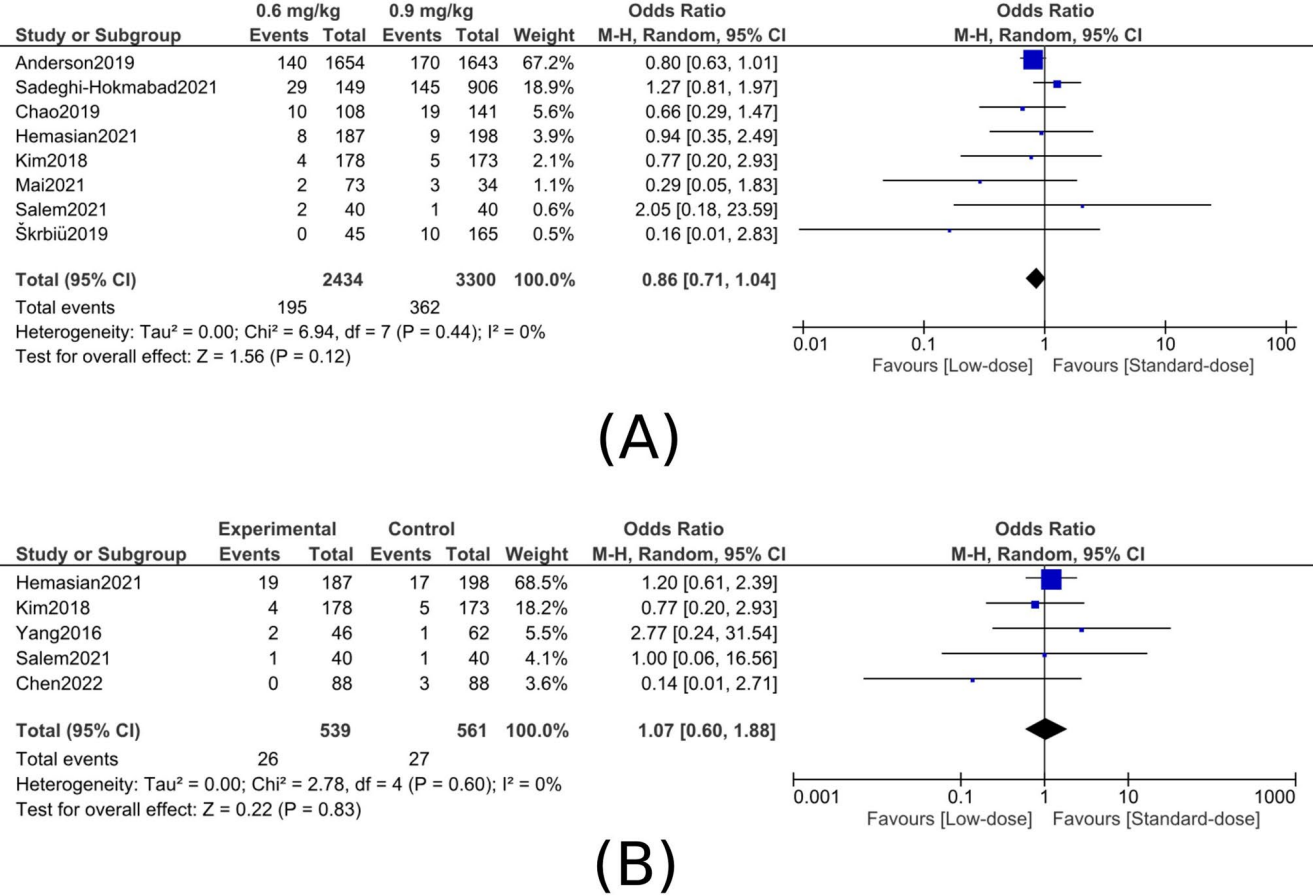
Comparison of low-dose (0.6 mg/kg) versus standard-dose (0.9 mg/kg) alteplase for mortality outcomes. **A**, 90-day mortality. **B**, In-hospital mortality
  B In-hospital mortality: Five studies (1,100 patients) found no significant difference between low- and standard-dose alteplase (OR, 1.07; 95% CI, 0.60–1.88; P = 0.83), with no heterogeneity detected (I² = 0%) (figure 3B).
2. Functional outcomes
  A Functional independence (mRS 0–1 at 90 days):Across six studies (5,099 patients), there was no significant difference between low- and standard-dose alteplase for achieving mRS 0–1 (OR, 0.90; 95% CI, 0.80–1.01; P = 0.09), with no heterogeneity (I² = 0%) (figure 4A).

**Fig. 4:**
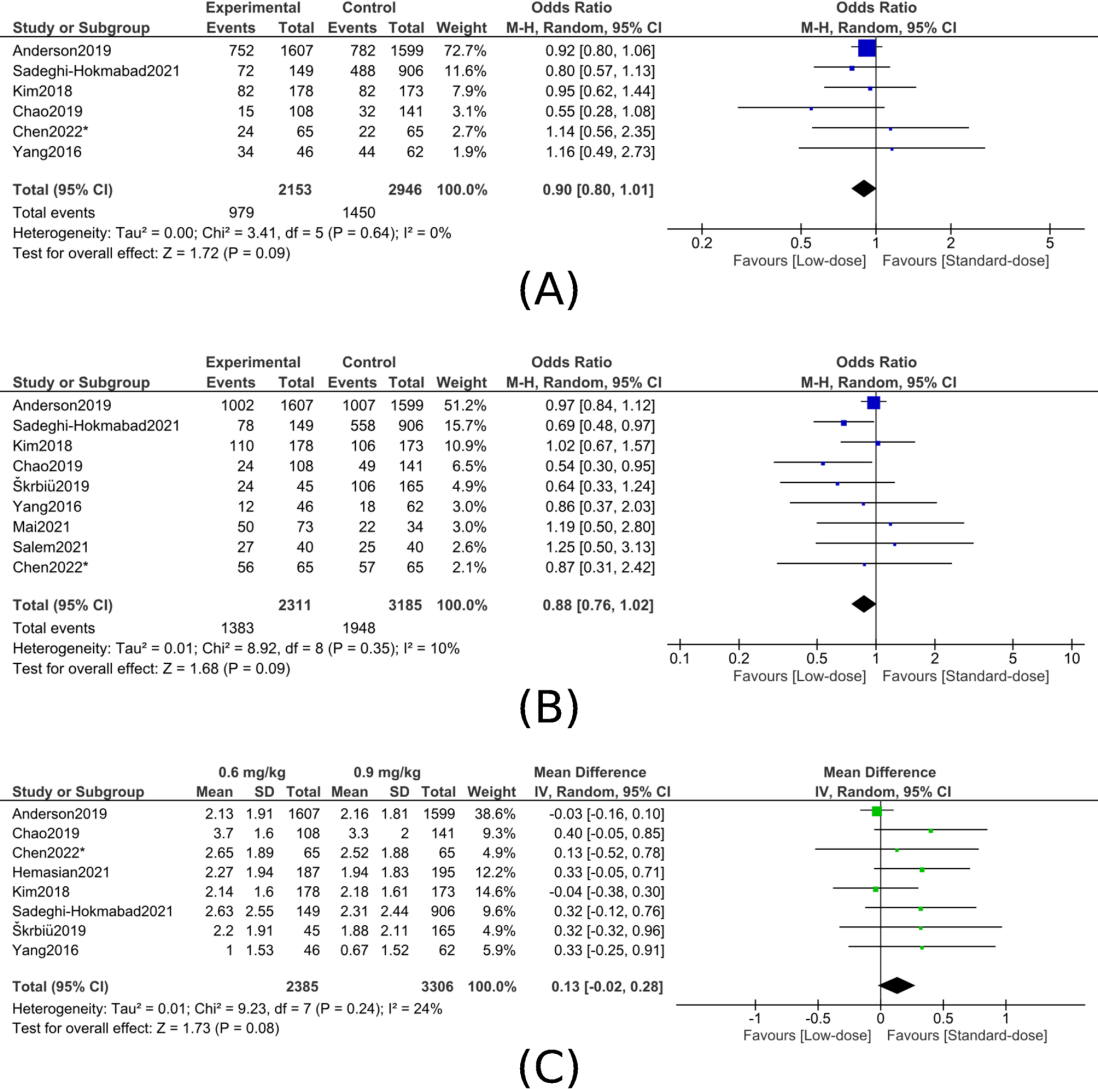
Comparison of low-dose (0.6 mg/kg) versus standard-dose (0.9 mg/kg) alteplase for functional outcomes at 90 days. **A**, Excellent functional outcome (mRS 0–1). **B**, Favorable functional outcome (mRS 0–2). **C**, Mean modified Rankin Scale (mRS) scores
  B Functional independence (mRS 0–2 at 90 days):Nine studies (5,496 patients) showed no significant difference between low- and standard-dose alteplase for mRS 0–2 (OR, 0.88; 95% CI, 0.76–1.02; P = 0.09), with low, non-significant heterogeneity (I²= 10%) (figure 4B).
  C amRS at 90 days (continuous): Eight studies (5,691 patients) found no significant difference in mean mRS between low- and standard-dose alteplase (mean difference, 0.13; 95% CI, −0.02 to 0.28; P = 0.08), with moderate, non-significant heterogeneity (I² = 24%) (figure 4C).

### Publication bias

We could not test for publication bias using Egger’s test since we did not include at least 10 studies for any of the outcomes, which is necessary to obtain accurate results [24].

### Risk of bias

**Fig. 5:**
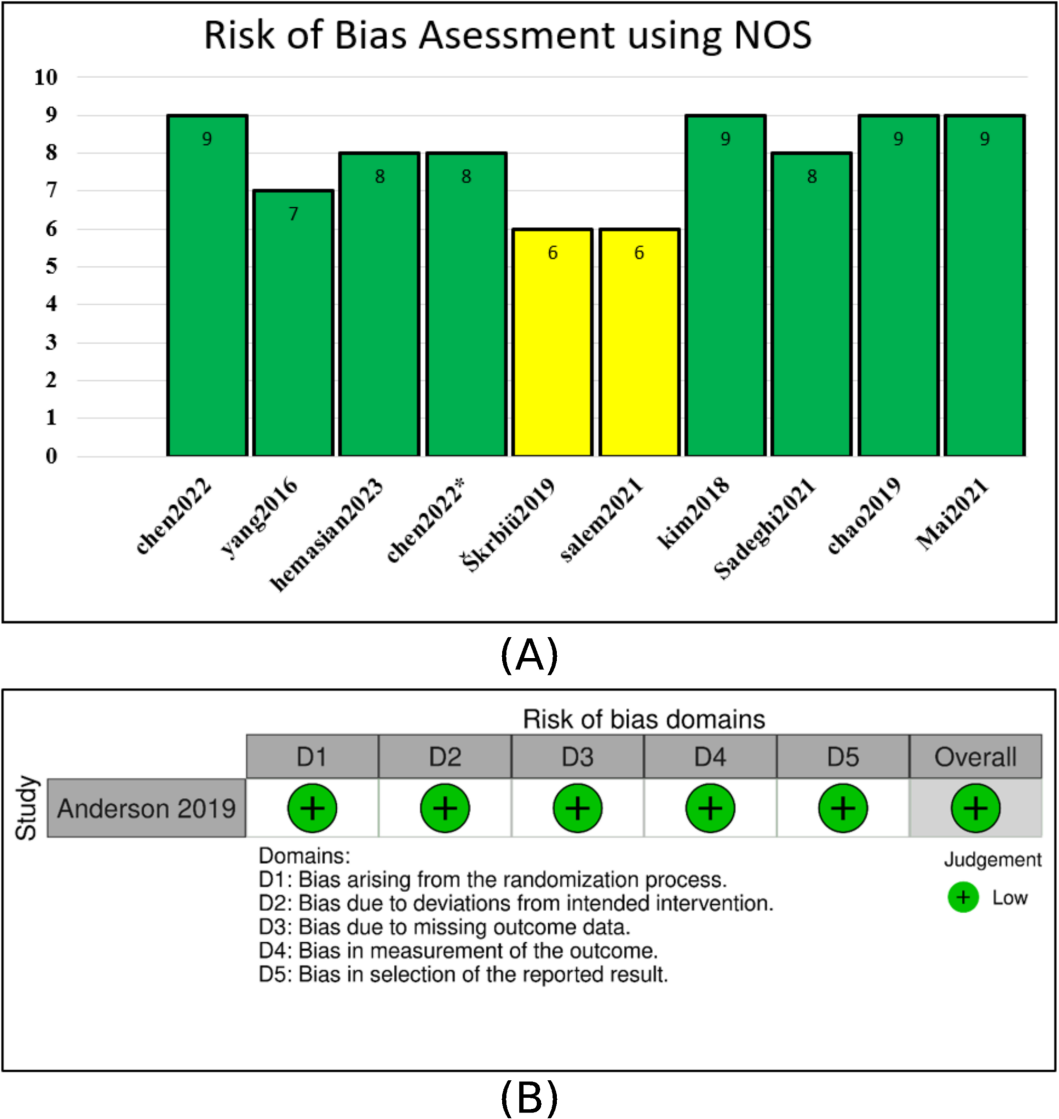
Risk of bias assessment for included studies. **A** Non-randomized study was evaluated using the Newcastle-Ottawa Scale (NOS). **B**, Randomized clinical trials evaluated using the RoB-2 tool

All included cohort studies were assessed using the Newcastle-Ottawa Scale (NOS), with most showing a low risk of bias. However, two studies [16] and [22] were rated as having a moderate risk as detailed in the supplementary file, Table S2, and illustrated in Fig. 5A. For randomized controlled trials, the revised Cochrane Risk of Bias tool (RoB 2) was used [5]. was judged to have a low risk of bias across all domains as illustrated in Fig. 5B.

### Strength of the evidence

The results of the strength of the evidence according to GRADE are summarized in Supplementary Table 3. The GRADE system classified the strength of evidence as moderate-certainty evidence for sICH. Low-dose alteplase was linked to a lower risk of sICH (OR 0.51; 95% CI, 0.35–0.76), supported by the large and consistent effect. For other outcomes—any ICH, 90-day mortality, functional independence (mRS 0–1 or 0–2), In-hospital mortality, and overall mRS scores, results slightly favored the standard dose but were not statistically significant. These findings were based on low-certainty evidence, as shown in the supplementary file, Table S3.

## Discussion

Our systematic review and meta-analysis studied the efficacy and safety of low-dose alteplase in adult patients with ischemic stroke. 11 studies with a total of 6,148 patients were included to evaluate sICH, any ICH, 90-day mortality, in-hospital mortality, and functional outcomes, which were measured by mRS.

According to our pooled estimates, low-dose alteplase was associated with significantly lower odds of sICH compared with standard dose (OR, 0.51; 95% CI, 0.35–0.76; *P* = 0.0008). The observed reduction in sICH with 0.6 mg/kg alteplase suggests a potential safety advantage in patients at elevated bleeding risk; however, this finding should be interpreted cautiously and requires prospective validation.

Notably, we did not find a statistically significant difference in the rate of any ICH (OR 1.00, 95% CI 0.84–1.19, *p* = 0.99) between the two doses, which suggests a similar risk of developing any ICH. These findings may indicate that patients treated with the low dose experienced less clinically severe ICH. These findings can be explained by the difference of definition between sICH and any ICH; sICH definitions (ECASS, SITS-MOST, NINDS) requires both radiological evidence of hemorrhage and clinical deterioration while sICH is more stringent and clinically relevant, this may indicate that while the overall incidence of hemorrhage remains similar, the severity and clinical impact of these hemorrhages are reduced with the low-dose. Also, timing and detection methods of hemorrhage may influence these results. sICH typically occurs within the first 24–36 h and is clinically apparent, while any ICH includes asymptomatic hemorrhages that may be detected on routine imaging at various time points. However, further research with detailed hemorrhage classification and severity scoring is required to explore the relationship between ICH severity, dose reduction, and clinical outcomes.

Also, we did not find a statistically significant difference in 90-day mortality (OR, 0.86; 95% CI, 0.71–1.04; *P* = 0.12). Similarly, the two dosing regimens did not show a statistically significant difference in in-hospital mortality (OR, 1.07; 95% CI, 0.60–1.88; *P* = 0.83). These findings suggest that the alteplase dose may not be a determining factor of mortality rates, and that other factors are more influential, such as time to treat, baseline prognostic factors, advanced age, and other co-morbid conditions [25–27].

Regarding functional recovery at 90 days, no statistically significant difference between the two doses was found in patients achieving excellent functional outcomes (mRS 0–1), favorable functional outcomes (mRS 0–2), and mean mRS scores. These results suggest that the low dose may offer similar efficacy to the standard dose, and that it could be preferred as a safer option in patients at high risk of bleeding.

A meta-analysis was published in 2017, suggested that for Asian patients with AIS, low-dose intravenous alteplase (0.6 mg/kg) offers a significant safety advantage in terms of reducing NINDS-defined sICH compared to the standard dose (0.9 mg/kg) (OR = 0.79, 95% CI 0.64–0.99, *p* = 0.04), without a significant overall difference in 90-day functional independence or mortality [28]. Furthermore, a prior meta-analysis in 2015 found no significant differences between low- and standard-dose intravenous alteplase for excellent functional outcome, sICH, or 3-month mortality. However, this review did not contain RCTs and used different definitions for low-dose (< 0.85 mg/kg) and standard dose (0.85–0.95.85.95 mg/kg) [29].

Importantly, the ENCHANTED trial, which was a large, international, randomized clinical trial on patients with AIS treated with alteplase, demonstrated that low-dose alteplase reduced the risk of sICH compared to the standard dose, with consistent results across age and ethnicity subgroups [5]. Those results are relatively aligned with our pooled results in terms of sICH.

This systematic review has several strengths. Including a comprehensive search, prespecified outcomes, and subgroup analyses according to established sICH definitions (ECASS II/III, SITS-MOST, NINDS). We also prioritized propensity score-matched estimates where available to reduce confounding. Furthermore, using the GRADE tool to assess the certainty of evidence for the outcomes. These design features increase the reliability of our estimates, but they do not eliminate inherent limitations of the underlying observational data.

However, several limitations should be considered. First, most of the included studies are observational in nature (10 of the included 11) and are subject to inherent bias, such as selection and confounding biases, due to the lack of randomization. Second, not all studies were included in every analysis; the number ranged from five to nine included studies for the different outcomes, which limits the statistical power for some outcomes (e.g., in-hospital mortality). Due to this variation, we couldn’t reliably assess the studies for publication bias using Egger’s test. Third, the included studies reported outcomes only up to 90 days post-treatment, and longer-term outcomes were not assessed, which limits the understanding of how the two doses of alteplase compare beyond 90 days. Also, A key limitation of this study is that the majority of included populations are Asian, which may limit the generalizability of our findings to other ethnic groups. Differences in genetics, body composition, and baseline stroke characteristics between Asian and non-Asian populations may affect how alteplase works and its safety. Future studies should specifically examine the dose-response relationship of alteplase in diverse ethnic populations. Finally, Endovascular thrombectomy (EVT) was variably reported and could be an important confounder of both hemorrhagic and functional outcomes. Because most studies did not provide outcome data stratified by EVT, subgroup analyses by EVT status were not feasible; this limitation is noted and highlights the need for future analyses reporting thrombolysis effects conditional on EVT.

Future work should examine effect modification by age, baseline NIHSS, onset-to-treatment time, EVT status, and ethnicity in stratified prospective studies. Ultimately, large randomized trials or prospective registries with standardized imaging protocols and harmonized sICH definitions are needed to confirm whether lowering the alteplase dose reduces clinically important hemorrhage while preserving efficacy.

## Conclusion

The 0.6 mg/kg alteplase dose significantly reduces sICH risk compared to 0.9 mg/kg in acute ischemic stroke, with no significant differences in functional outcomes or mortality. These findings suggest potential safety benefits of lower-dose therapy, warranting further investigation in future studies.

## Supplementary Information

Supplementary Material 1. Supplementary file: Table 1: Search strategy for each database. Supplementary file: Table 2: Detailed risk of bias assessment using NOS for cohort studies. Supplementary file: Table 3: GRADE assessment for each outcome. Supplementary file: Figure 1: subgroup analysis for sICH by each diagnostic criterion. 

Supplementary Material 2.

## Data Availability

Not applicable.

## Abbreviations

AIS: Acute ischemic stroke
CE: Cardioembolism
CI: Confidence interval
CT: Computed tomography
ECASS: European Cooperative Acute Stroke Study
ENCHANTED: Enhanced Control of Hypertension and Thrombolysis Stroke Study
EVT: Endovascular therapy
GRADE: Grading of Recommendations, Assessment, Development, and Evaluation
ICH: Intracranial hemorrhage
IV: Intravenous
LAA: Large-artery atherosclerosis
MD: Mean difference
MRI: Magnetic resonance imaging
mRS: Modified Rankin Scale
NIHSS: National Institutes of Health Stroke Scale
NINDS: National Institute of Neurological Disorders and Stroke
NOS: Newcastle–Ottawa Scale
OR: Odds ratio
PMS: Propensity-matched studies
PRISMA: Preferred Reporting Items for Systematic Reviews and Meta-Analyses
RCT: Randomized controlled trial
RoB2: Risk of Bias 2 tool
SD: Standard deviation
sICH: Symptomatic intracranial hemorrhage
SITS-MOST: Safe Implementation of Thrombolysis in Stroke-Monitoring Study
SVO: Small-vessel occlusion
TOAST: Trial of Org 10172 in Acute Stroke Treatment
