# Supplementary material for: Comparative safety and efficacy of 0.6 mg/kg versus 0.9 mg/kg alteplase in acute ischemic stroke: a systematic review and meta-analysis
Source: BMC Neurol. 2025 Nov 18;25:471. doi: 10.1186/s12883-025-04481-1 (PMC12625435; doi:10.1186/s12883-025-04481-1)
Supplement: Supplementary file 1 — Supplementary Material 1. Supplementary file: Table 1: Search strategy for each database. Supplementary file: Table 2: Detailed risk of bias assessment using NOS for cohort studies. Supplementary file: Table 3: GRADE assessment for each outcome. Supplementary file: Figure 1: subgroup analysis for sICH by each diagnostic criterion. [file 12883_2025_4481_MOESM1_ESM.docx]

| **Table 1: Search strategy for each database** | | |
| --- | --- | --- |
| **Database** | **Search strategy** | **results** |
| **PubMed** | ("ischemic stroke" OR "acute ischemic stroke" OR "acute brain ischemia" OR "thrombotic stroke" OR "embolic stroke" OR "cerebral embolism" OR "cerebral thrombosis" OR "large vessel occlusion stroke" OR "LVO stroke" OR "small vessel ischemic stroke" OR "SVI" OR "wake-up stroke" OR "atherothrombotic stroke") AND ("low dose" AND (alteplase OR rTPA OR tPA) OR "reduced dose" AND (alteplase OR rTPA OR tPA) OR "0.6 mg/kg" AND (alteplase OR rTPA OR tPA)) AND ("standard dose" AND (alteplase OR rTPA OR tPA) OR "0.9 mg/kg" AND (alteplase OR rTPA OR tPA) OR "full dose" AND (alteplase OR rTPA OR tPA) OR "high dose" AND (alteplase OR rTPA OR tPA)) | 113 |
| **Web of science** | TS=("ischemic stroke" OR "acute ischemic stroke" OR "acute brain ischemia" OR "thrombotic stroke" OR "embolic stroke" OR "cerebral embolism" OR "cerebral thrombosis" OR "large vessel occlusion stroke" OR "LVO stroke" OR "small vessel ischemic stroke" OR "SVI" OR "wake-up stroke" OR "atherothrombotic stroke")  AND  TS=("low dose" AND (alteplase OR rTPA OR tPA) OR "reduced dose" AND (alteplase OR rTPA OR tPA) OR "0.6 mg/kg" AND (alteplase OR rTPA OR tPA))  AND  TS=("standard dose" AND (alteplase OR rTPA OR tPA) OR "0.9 mg/kg" AND (alteplase OR rTPA OR tPA) OR "full dose" AND (alteplase OR rTPA OR tPA) OR "high dose" AND (alteplase OR rTPA OR tPA)) | 117 |
| **Scopus** | TITLE-ABS-KEY ( "ischemic stroke" AND ( "low dose" OR "0.6 mg/kg" ) AND ( "standard dose" OR "0.9 mg/kg" ) AND ( alteplase OR rTPA OR tPA ) AND ( comparison OR vs OR versus ) ) | 79 |

| **Table 2: the detailed risk of bias assessment using NOS for cohort studies** | | | | | | | | | |
| --- | --- | --- | --- | --- | --- | --- | --- | --- | --- |
| **StudyID** | **Representativeness of the exposed cohort** | **Selection of the non-exposed cohort** | **Ascertainment of exposure** | **Demonstration that outcome of interest was not present at start of study** | **Control for important or additional factors** | **Assessment of outcome** | **Was follow-up long enough for outcomes to occur** | **Adequacy of follow up of cohorts** | **Total score** |
| **chen2022** | 1 | 1 | 1 | 1 | 2 | 1 | 1 | 1 | 9 |
| **yang2016** | 0 | 1 | 1 | 1 | 1 | 1 | 1 | 1 | 7 |
| **hemasian2023** | 1 | 1 | 1 | 1 | 1 | 1 | 1 | 1 | 8 |
| **chen2022*** | 0 | 1 | 1 | 1 | 2 | 1 | 1 | 1 | 8 |
| **Škrbiü2019** | 0 | 1 | 1 | 1 | 0 | 1 | 1 | 1 | 6 |
| **salem2021** | 0 | 1 | 1 | 1 | 0 | 1 | 1 | 1 | 6 |
| **kim2018** | 1 | 1 | 1 | 1 | 2 | 1 | 1 | 1 | 9 |
| **Sadeghi2021** | 0 | 1 | 1 | 1 | 2 | 1 | 1 | 1 | 8 |
| **chao2019** | 1 | 1 | 1 | 1 | 2 | 1 | 1 | 1 | 9 |
| **Mai2021** | 1 | 1 | 1 | 1 | 2 | 1 | 1 | 1 | 9 |

| **Table 3: GRADE assessment for each outcome** | | | | | | | | | | | | |
| --- | --- | --- | --- | --- | --- | --- | --- | --- | --- | --- | --- | --- |
| **Certainty assessment** | | | | | | | **№ of patients** | | **Effect** | | **Certainty** | **Importance** |
| **№ of studies** | **Study design** | **Risk of bias** | **Inconsistency** | **Indirectness** | **Imprecision** | **Other considerations** | **Low-dose** | **Standard-dose** | **Relative (95% CI)** | **Absolute (95% CI)** |  |  |
| **sICH** | | | | | | | | | | | | |
| 9 | non-randomised studies | not serious | not serious | not serious | not serious | strong association | 39/2401 (1.6%) | 90/3152 (2.9%) | **OR 0.51** (0.35 to 0.76) | **14 fewer per 1,000** (from 18 fewer to 7 fewer) | ⨁⨁⨁◯ Moderate | CRITICAL |
| **Any ICH** | | | | | | | | | | | | |
| 8 | non-randomised studies | not serious | not serious | not serious | not serious | none | 96/784 (12.2%) | 131/904 (14.5%) | **OR 1.00** (0.84 to 1.19) | **0 fewer per 1,000** (from 20 fewer to 23 more) | ⨁⨁◯◯ Low | CRITICAL |
| **90-day mortality:** | | | | | | | | | | | | |
| 8 | non-randomised studies | not serious | not serious | not serious | not serious | none | 195/2434 (8.0%) | 362/3300 (11.0%) | **OR 0.86** (0.71 to 1.04) | **14 fewer per 1,000** (from 29 fewer to 4 more) | ⨁⨁◯◯ Low | CRITICAL |
| **Functional independence (mRS 0–1 at 90 days)** | | | | | | | | | | | | |
| 6 | non-randomised studies | not serious | not serious | not serious | not serious | none | 979/2153 (45.5%) | 1450/2946 (49.2%) | **OR 0.90** (0.80 to 1.01) | **26 fewer per 1,000** (from 55 fewer to 2 more) | ⨁⨁◯◯ Low | IMPORTANT |
| **New outcome Functional independence (mRS 0–2 at 90 days)** | | | | | | | | | | | | |
| 9 | non-randomised studies | not serious | not serious | not serious | not serious | none | 1383/2311 (59.8%) | 1948/3185 (61.2%) | **OR 0.88** (0.76 to 1.02) | **31 fewer per 1,000** (from 67 fewer to 5 more) | ⨁⨁◯◯ Low | CRITICAL |
| **In-hospital mortality** | | | | | | | | | | | | |
| 5 | non-randomised studies | not serious | not serious | not serious | not serious | none | 26/539 (4.8%) | 27/561 (4.8%) | **OR 1.07** (0.60 to 1.88) | **3 more per 1,000** (from 19 fewer to 39 more) | ⨁⨁◯◯ Low | CRITICAL |
| **Modified Rankin Scale (mRS) at 90 days** | | | | | | | | | | | | |
| 8 | non-randomised studies | not serious | not serious | not serious | not serious | none | 2385 | 3306 | - | MD **0.13 higher** (0.02 lower to 0.28 higher) | ⨁⨁◯◯ Low | IMPORTANT |

**CI:** confidence interval; **MD:** mean difference; **OR:** odds ratio


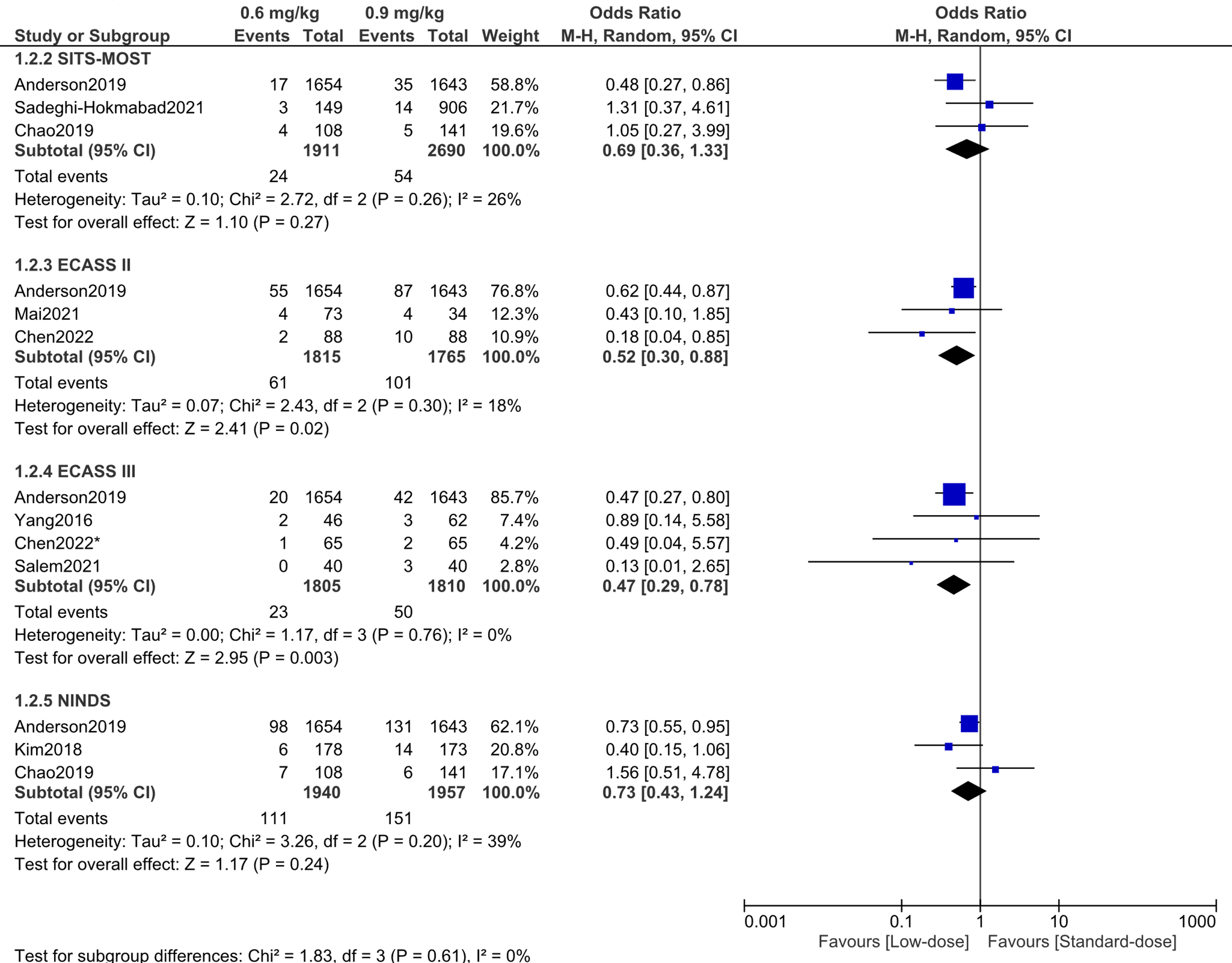


Figure 1: subgroup analysis for sICH by each diagnostic criterion.
